# Hardware implementation of EKF based SOC estimate for lithium-ion batteries in electric vehicle applications

**DOI:** 10.1038/s41598-025-99931-8

**Published:** 2025-05-03

**Authors:** G. Nishanth, M. Murali Krishnan, Balamurugan Parandhaman, J. Harinarayanan

**Affiliations:** School of Electrical Engineering, Vellore Institute of Technology, Chennai, India

**Keywords:** State of charge, Extended Kalman filter, Lithium-ion battery, Electric vehicle, Equivalent circuit model, Energy science and technology, Engineering, Electrical and electronic engineering

## Abstract

In the rechargeable batteries lithium-ion batteries are now being utilized extensively in a variety of industries, including electric vehicles, drones, and portable electronics. When it comes to such batteries, it is extremely challenging to accurately monitor the state of charge (SOC). In this instance, the EKF method has been implemented with software and hardware demonstration, and the measured value and estimated value have both achieved an error that is within 2% of each other. As a result of executing the static capacity test under dV/dt with the discharging current set to constant and hybrid pulse power characterization test, the figures that are becoming available are being acquired. The approach of optimization assigns a SOC of 90% and 10% for two reference points in the V_cell_ equation. Additionally, the technique updates the electrical model of the cell by using the derivative of the terminal voltage that was recorded for the cell. With the use of the covariance matrices in the Extended Kalman Filtering equations, the SOC of the battery may be reliably predicted with a level of accuracy that exceeds 98% when compared to conventional techniques of estimation such as coulomb counting. As part of this research, an adaptive model approach for evaluating the state of charge of Li-ion batteries that are becoming older is being developed.

## Introduction

Electric vehicles (EVs) have emerged as a promising solution to reduce greenhouse gas emissions and dependency on fossil fuels. In India, the adoption of electric vehicles has been on a steady rise, with a remarkable year-on-year growth of 82% from March 2022 to 2023^1^. However, the massive population and the extensive use of vehicles in the country underscore the importance of effectively managing the lifecycle of EV batteries to maximize their utility and minimize premature scrapping. A crucial aspect of this management lies in accurately estimating the SOC of batteries throughout their lifespan. Various methods, such as coulomb counting, Ampere-hour method, neural network method, and open circuit voltage method, have been employed for SOC estimation^2^. While these methods offer different advantages, they also come with their limitations, particularly in maintaining accuracy over time. One method that has shown promise in addressing these challenges is the Kalman filter, particularly its nonlinear variant, the Extended Kalman Filter (EKF)^3^.

As batteries age, they undergo chemical changes that affect their SOC differently. This variation poses a challenge for traditional SOC estimation methods, which may struggle to accurately account for these changes. The Ampere-hour method, for instance, initially provides high accuracy but suffers from cumulative errors over time, leading to decreased accuracy with prolonged operation. These limitations necessitate the development of adaptive methods that can dynamically adjust to real-time corrections in SOC estimation^4^.

Kalman filters, widely used in control systems and signal processing, offer a powerful framework for state estimation in dynamic systems. The Extended Kalman Filter, in particular, extends the basic Kalman filter to nonlinear systems by linearizing the mean and covariance^5^. This nonlinear variant proves to be particularly effective in addressing the complexities associated with battery aging and SOC estimation. The EKF introduces a time-dependent update mechanism for its matrices to account for the changing behaviour of batteries over time. As batteries age, their characteristics evolve, necessitating adjustments in the estimation process. By updating the EKF matrices with equations that capture these time-dependent changes, the filter can adapt to the evolving dynamics of battery behaviour^6^.

Despite its effectiveness, the implementation of EKF for battery management poses several challenges. Nonlinearities in battery behaviour, measurement noise, and computational complexity are among the key challenges that need to be addressed. Researchers have proposed various solutions to overcome these challenges, including improved modelling techniques, sensor fusion approaches, and optimization algorithms^7^. A novel deep learning technique for battery State of Charge estimation using labelled and unlabelled training data is presented in the study. Many labelled samples (current/voltage data with known SOC) are expensive to collect using traditional approaches. An input reconstruction network is added to the supervised network to retrieve meaningful information from unlabelled data. Their approach outperformed labelled samples by over 14% in various driving cycles and temperature conditions. The method works with LSTM, GRU, and BLSTM recurrent neural networks and is resilient to minimal labelled data^8^.

Recent advancements in battery monitoring systems have further enhanced the capabilities of EKF-based SOC estimation. Integration with advanced sensors, such as impedance spectroscopy and infrared thermography, enables more comprehensive monitoring of battery health and performance. Machine learning algorithms, coupled with EKF, offer enhanced predictive capabilities, enabling proactive maintenance and optimization strategies. The ongoing research and development in battery management systems are focused on improving the accuracy, reliability, and efficiency of SOC estimation. Integration with emerging technologies, such as Internet of Things (IoT) and cloud computing, holds promise for real-time monitoring and remote management of EV batteries. Additionally, advancements in materials science and battery chemistry are expected to yield batteries with improved performance and longevity, further enhancing the effectiveness of SOC estimation techniques.

Batteries come in various types, each designed for specific applications and offering unique characteristics. The most common types include alkaline batteries, known for their long shelf life and versatility in low-drain devices like remote controls; lithium-ion batteries, favoured for their high energy density and rechargeability, commonly used in smartphones, laptops, and electric vehicles; nickel-metal hydride (NiMH) batteries, offering a balance between capacity and cost, often found in cameras, toys, and power tools; lead-acid batteries, known for their robustness and ability to deliver high current, typically used in automotive, uninterruptible power supplies (UPS), and renewable energy storage systems; and nickel-cadmium (NiCd) batteries, valued for their durability and reliability, though less common due to environmental concerns, still utilized in applications like emergency lighting and cordless power tools. Each type has its advantages and limitations, catering to a wide range of consumer and industrial needs.

SOC and State of Health (SOH) are important parameters used to assess the condition and performance of batteries. SOC indicates the amount of energy remaining in a battery relative to its full capacity. It is usually expressed as a percentage. Knowing the SOC helps users estimate how much runtime is left before the battery needs recharging. SOH reflects the overall condition and performance capability of a battery compared to its original condition when new. It considers factors such as capacity degradation, internal resistance increase, and other aging effects. SOH provides insights into a battery’s remaining lifespan and its ability to deliver the required performance. Both SOC and SOH are crucial for managing and maintaining battery systems effectively, ensuring optimal performance, and preventing unexpected failures. They are utilized in various applications, including electric vehicles, renewable energy storage systems, portable electronics, and grid-scale energy storage.

In this study the SOC estimation is carried out in three phases: phase-I conduct the static capacity test and hybrid pulse power characterisation (HPPC) test, phase-II develops hardware to estimate the SOC using the EKF, and the final phase validates the findings of both software and hardware. Throughout this test, a Samsung cell 18,650, 2.6Ah, 3.6 V NMC cell was used throughout the whole procedure at room temperature of 25 °C with 1 C rate for both SCT and the HPPC test.

## Extended Kalman filter

Kalman Filter is a famous method of estimation that breaks down the problem to smaller bits and by linear filtering it estimates the state variables. It handles linear and discrete systems that are time dependent. By calculating minimum mean squared error estimate of current state Kalman filter finds unknown variables.

x_k+1_ = A_k_ x_k_ + B_k_u_k_ + w_k_.

y_k_ = C_k_x_k_ + D_k_u_k_ + w_k_.

where u_k_ = input to the system, w_k_ = process noise, y_k_ = output of the system, v_k_ = measurement noises, x_k_ = system state vector at time k, x_k+1_ = system state vector at time k + 1, A_k_, B_k_, C_k,_ D_k_ = matrices describing dynamics of the system.

Kalman filter follows prediction and correction methodology where in prediction using the model it predicts current state, covariance and system output. At correction step, with use of an actual measurement the filter further improves estimated state and the error covariance.

The filter goes in a cycle of steps from Prior knowledge → Prediction → Update (with measurement inputs) → Next time step/Output → Prediction (next time step).

To combat nonlinear equations Extended Kalman filter is introduced, which linearizes the equations at each time step giving a linear time varying system which can be used in Kalman filter.

x_k+1_ = *f*(x_k_, u_k_) + w_k_

 y_k_ = g(x_k_, u_k_ )+ v_k_.

Where *f* = transition function of system, *u*_*k*_ = control signal, *w*_*k*_,*v*_*k*_ = zero mean white gaussian stochastic process, *x*_*k*_ = system state vector at time k, *x*_*k+1*_ = system state vector at time k + 1,

at each time step,* f*(x_k_, u_k_ ) and* g*(x_k_, u_k_ ) get linearized by using 1st order Taylor series.

Input.

1. Initial estimates of $\:{\widehat{x}}_{k}-1$ and $\:{\widehat{P}}_{k}-1$

Time Update (‘Predict’).

(2) Next state: $$\:{\widehat{X}}_{k}^{-}=A{\widehat{X}}_{k-1}+B{u}_{k}$$
(3) Next state error covariance: P_k_^−^ = AP_k−1_A^T^ + Q.

Measurement update (‘Correct’).

1. Calculate Kalman gain: K_k_ = P_k_−C^T^ (CP_k_^−^C^T^ + R)^−1^.
2. Update estimates with measurement y_k_: $\:{\widehat{X}}_{k}={\widehat{X}}_{k}^{-}$+ K_k_ ($\:{y}_{k}$-C$\:{\widehat{X}}_{k}^{-}$)

Update error covariance: P_k_ = (I – K_k_C) P_k_^−^

where $\:{\widehat{X}}_{k}^{-}$ = predict state estimate, P_k_^−^ = predict state covariance, K_k_ = Kalman gain matrix, P_k_ = correct state covariance, $\:{\widehat{x}}_{k}$ = minimum squared error estimate

## Software implementation

The model is built and tested using MATLAB and Simulink software tools in which a battery model is created and given an error to mimic real life conditions that is nonlinear and random^9^.

### MATLAB coding

Set the initial values for the state vector (SOC and polarization voltage), the state error covariance matrix, the process noise, and the observation noise.

Where X-Initial state vector representing SOC and polarization voltage; P0-Initial state error covariance matrix; Q and R- Process and observation noise covariance; V_t_measured_ -Measured terminal voltage (initialized with zeros); I-Constant discharge current-B: Coulombic efficiency- C; _AH - Total battery capacity; Ts-Sampling time; N-Number of iterations.

**Fig. 1:**
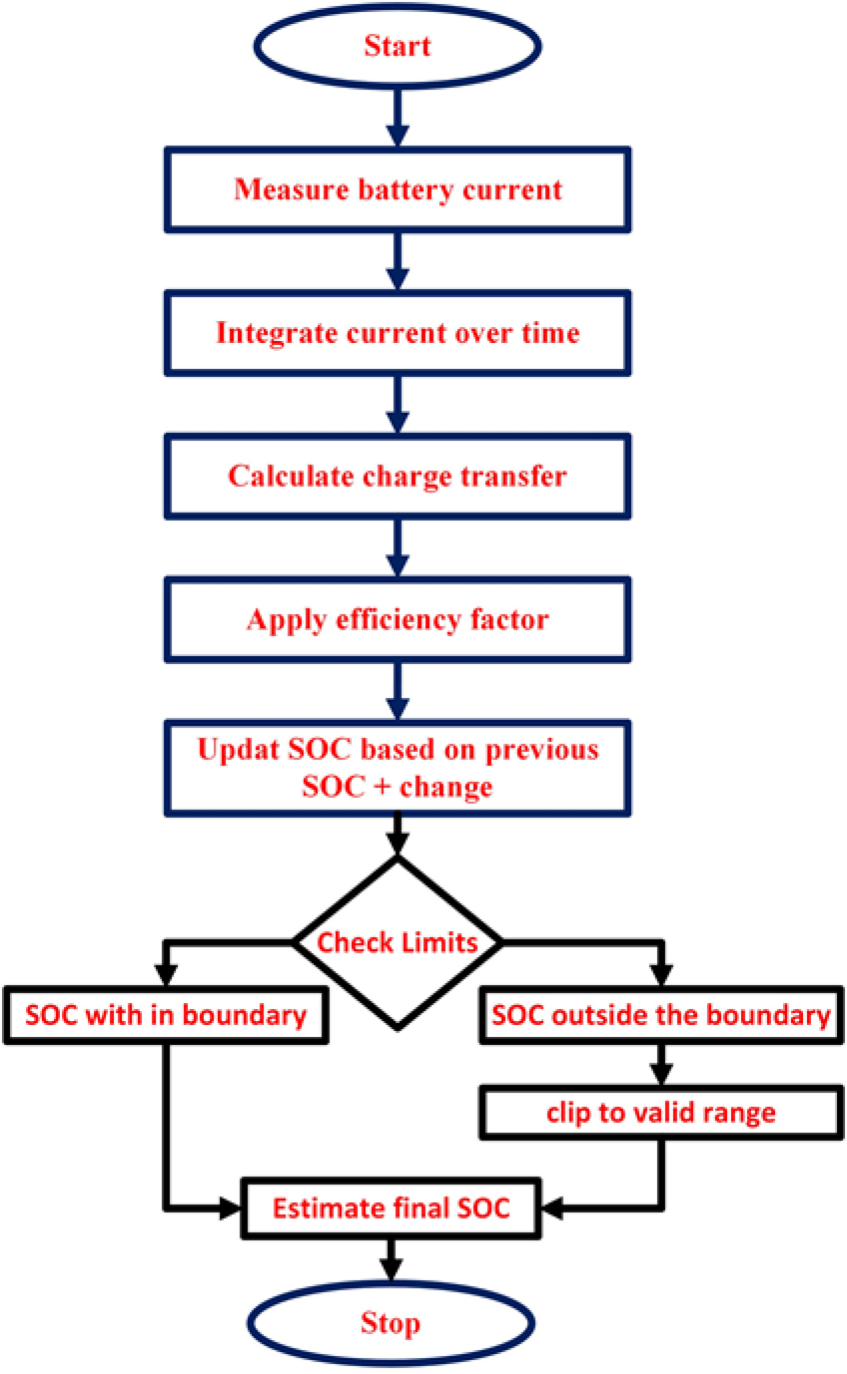
Flowchart of Coulomb Counting algorithm for SOC computation in MATLAB.

Simulate the true system by iterating over a loop that updates the SOC, internal resistance, polarization resistance, polarized capacitance, and terminal voltage at each time step, based on the current state and a constant discharge current. The terminal voltage is measured with noise. The various stages in the estimation of SOC using EKF using MATLAB is depicted as flowchart in Fig. 1.

Where SOC_initial: Retrieves the SOC from the previous time step. R0, Rp, Cp are calculated internal resistance, polarization resistance, and polarized capacitance based on the current SOC. Tao is the time constant calculated as the product of polarization resistance (Rp) and polarized capacitance (Cp. A: State transition matrix that predicts the state for the next time step. B: Input control matrix that considers the effect of the discharge current. X(:, t) Updates the state vector (SOC and polarization voltage) using the state transition matrix, input control matrix, and process noise. Open Circuit Voltage (Voc) is calculated based on a polynomial relationship with the SOC. Vt_measured (1, t) is used to calculate the measured terminal voltage considering Voc, Vp_initial, discharge current I, internal resistance R0, and observation noise.

So, this part of the code simulates the true values of the SOC and terminal voltage by updating the state vector based on a battery model and introducing noise in the measurements. The open circuit voltage and polarization voltage are calculated based on polynomial relationships, and the measured terminal voltage is obtained by considering various factors such as internal resistance and observation noise.

Implement the EKF algorithm by iterating over another loop that updates the state prediction and error covariance matrices using the state transition and observation models, and the predicted and measured terminal voltages. The state prediction is based on the previous state estimate and the current discharge current. The observation model calculates the expected terminal voltage based on the current state estimate.

**Fig. 2:**
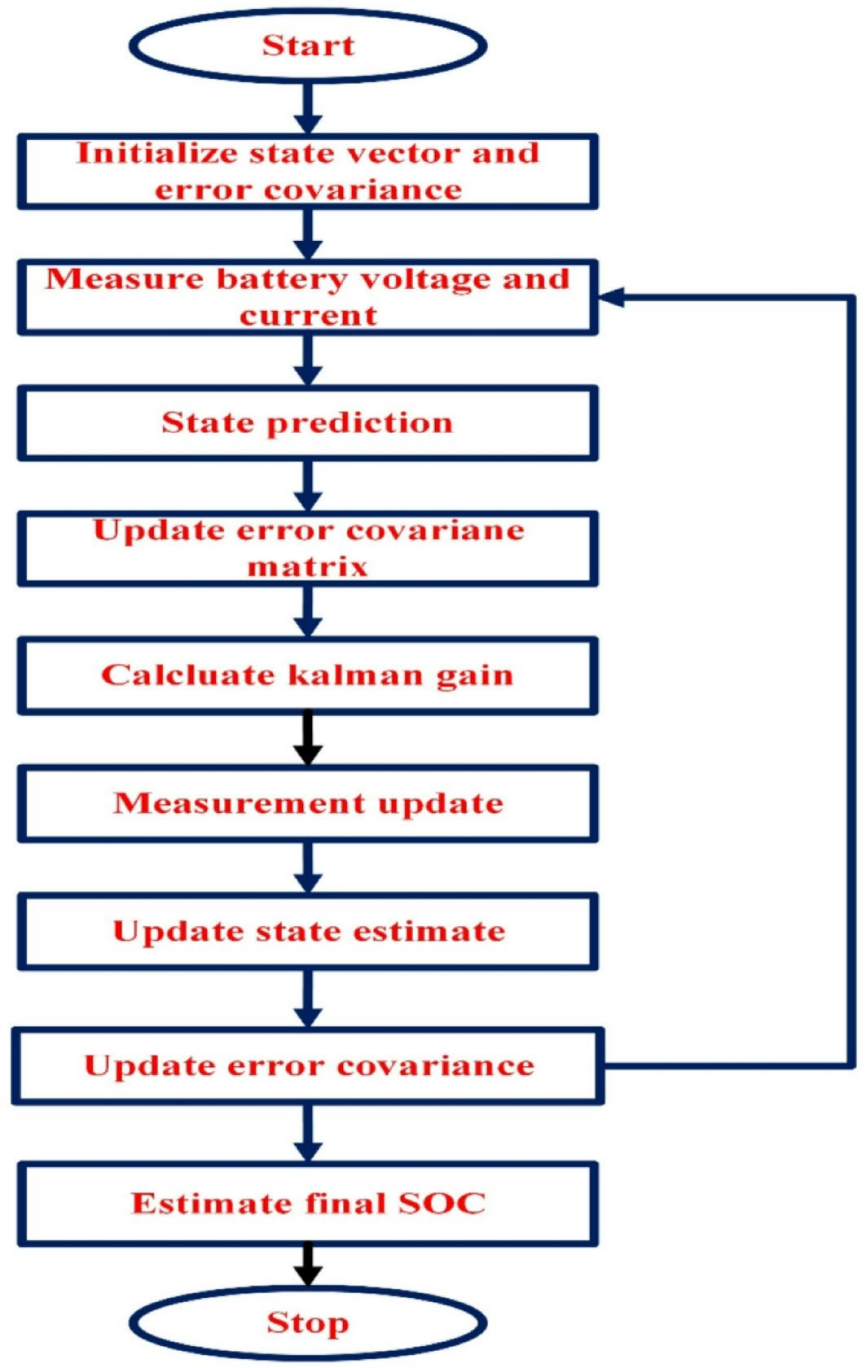
Flowchart of EKF algorithm for SOC computation in MATLAB.

Figure 2 shows an Extended Kalman Filter (EKF) algorithm is applied to estimate the state variables of a battery system, particularly the SOC and polarization voltage. The algorithm processes measurements of the terminal voltage of the battery (Vt_measured) and uses a battery model for prediction. The process begins by initializing the state vector (X) and the state error covariance matrix (P0). The battery model parameters, such as internal resistance (R0), polarization resistance (Rp), and polarized capacitance (Cp), are determined based on the current SOC. The time constant (Tao) is computed, and matrices (A and B) for the state transition are defined. The prediction step (X_predict) estimates the current state using the battery model and the discharge current (i). The predicted terminal voltage (Vt_estimated) is calculated by subtracting the polarization voltage (Vp_predict) from the open circuit voltage (Voc). Variance and covariance matrices are updated accordingly. The Kalman gain (K_gain) is computed based on the predicted SOC and used to update the state vector (X_update) by incorporating the difference between the measured and estimated terminal voltages. The state error covariance matrix (P0) is also updated. The code tracks and records the error percentage€) between the predicted SOC and the true SOC. The final result is a set of estimated states (X_update) and an error percentage cu€ (e).

**Fig. 3:**
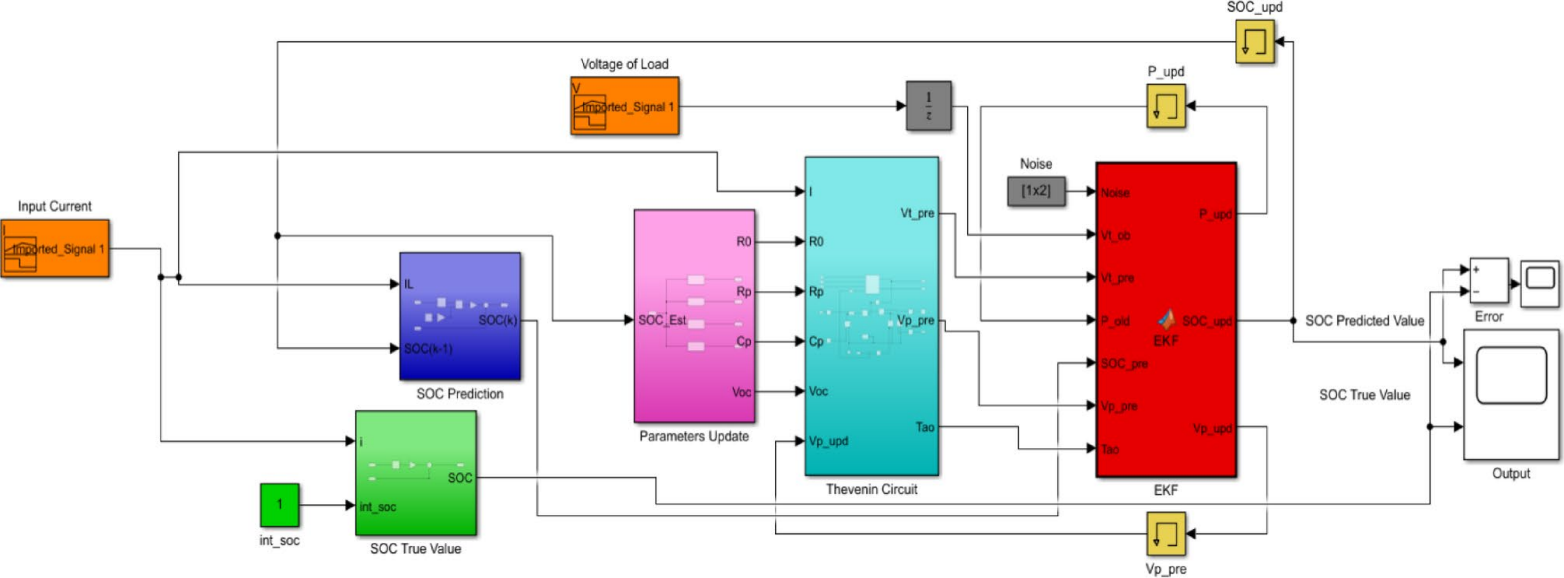
MATLAB simulink.

Here the Fig. 3 shows to visualize the results of an Extended Kalman Filter (EKF) applied to estimate SOC in a battery system. In the figure, two subplots are displayed vertically. The first subplot (top) illustrates the comparison between the EKF-predicted SOC values (depicted in blue) and the actual measured SOC values (depicted in red) over a specified time range (t = 1: N) as shown in Fig. 5. The x-axis represents time in seconds (t(s)), and the y-axis represents the SOC. The grid is enabled for better readability, and a legend indicates the colours for the EKF predicted and measured values. In the second subplot (bottom), the EKF error is plotted against time as shown. The x-axis represents time in seconds (t(s)), and the y-axis represents the error percentage (Error (%)). The error is calculated as the difference between the EKF-predicted SOC and the actual measured SOC, expressed as a percentage. The grid is enabled for clarity, and a legend denotes the color for the EKF error plot.

According to the results shown in Fig. 3, the difference between the SOC estimated by the Extended Kalman Filter (EKF) and the reference SOC calculated by coulomb counting would be nearly 3% without utilizing the updated electrical model for cells with DC motor load. This indicates EKF’s effectiveness in SOC estimation significantly depends on the availability of an updated electrical model for batteries.

Here two test conducted one static capacity test and hybrid pulse power characterization test. With those values are calculated with the help of Hybrid Pulse Power Characterisation (HPPC) test which is conducted in our lab. Those current and voltage values are further used in the SOC prediction using Extended Kalman Filter^10^. Here for comparison, a block called SOC true value has also been made which uses coulomb counting method to find the SOC value.

High Power Pulse Capability (HPPC) testing is a crucial evaluation method for Lithium-Ion (Li-ion) batteries, such as the popular 18,650 cell format. The HPPC test involves subjecting the battery to high-rate charging and discharging pulses to assess its dynamic performance under varying loads and conditions. This test is particularly relevant for applications requiring rapid power delivery, such as electric vehicles and high-performance electronics. During an HPPC test, the Li-ion battery undergoes cycles of high-current charging and discharging pulses^11^. These pulses mimic the rapid power demands that the battery may experience in real-world scenarios. The test evaluates the ’attery’s ability to deliver power efficiently without compromising its capacity, voltage, and thermal stability. It helps identify the dynamic response of the battery under different stress conditions, offering insights into its overall performance and suitability for high-power applications. Results from HPPC testing provide valuable data for battery management system optimization, design improvements, and ensuring the ’attery’s safe and reliable operation in demanding environments.

**Fig. 4:**
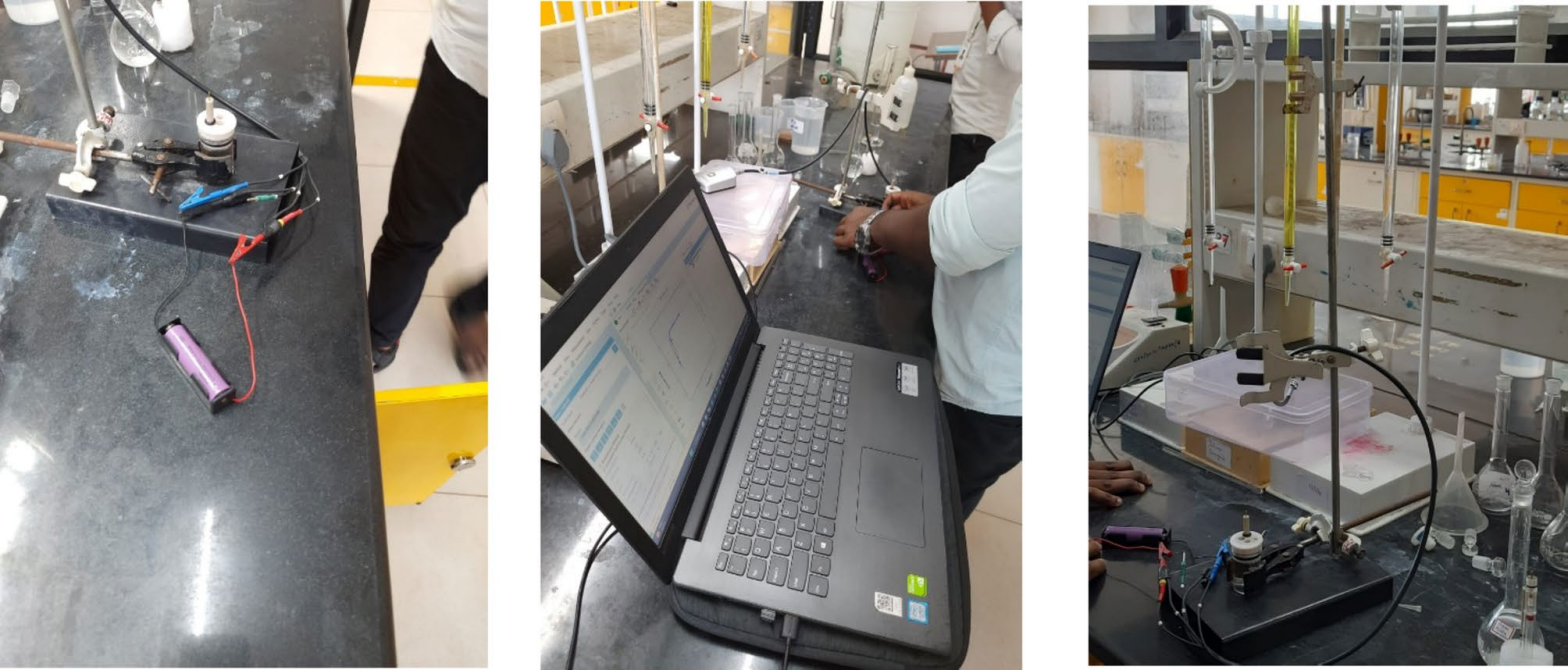
Hardware setup for HPPC test.

Figure 4 represents the experimental setup of the HPPC (Hybrid Pulse Power Characterization) test conducted by using the PalmSens potentiostat in PSTrace software allows for detailed analysis of a battery’s dynamic behavior under various pulse conditions. The first step is to connect the 18,650 Li-ion battery to the PalmSens device, ensuring the correct configuration of the Working, Counter, and Reference electrodes based on the test setup. Once connected, launch the PSTrace software, which will automatically detect the potentiostat. Select the device and configure the HPPC test, typically using either Chronoamperometry or the Galvanostatic Intermittent Titration Technique (GITT), depending on the specific analysis needs.

**Fig. 5:**
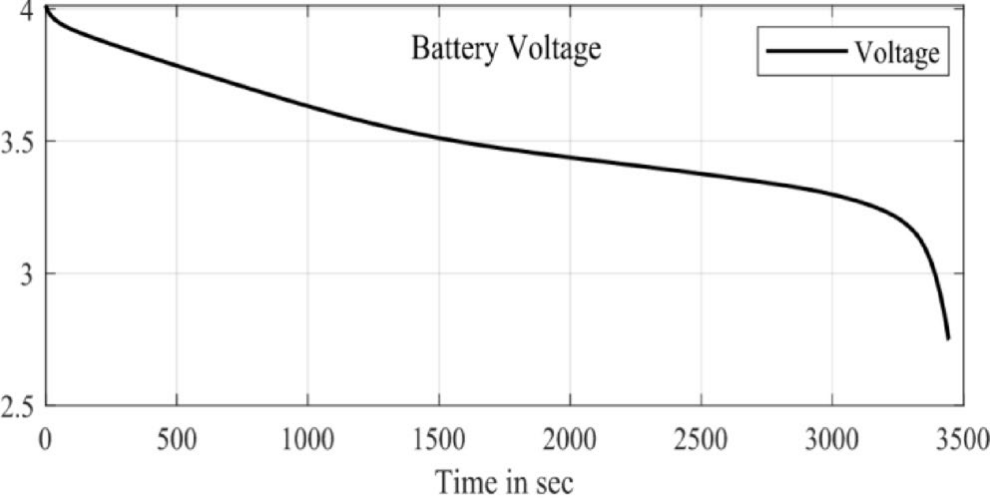
SCT Test discharge curve.

**Fig. 6:**
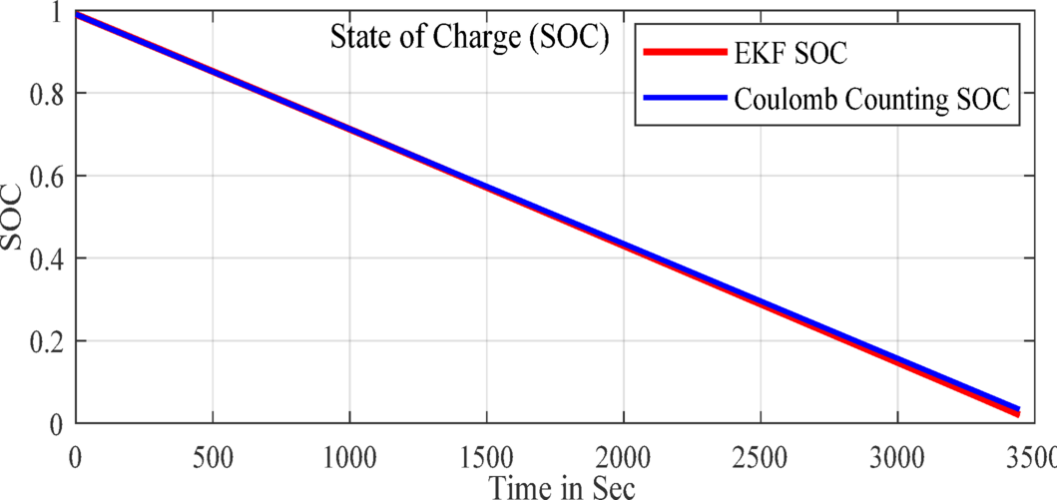
SOC estimated by EKF and CC methods.

**Fig. 7:**
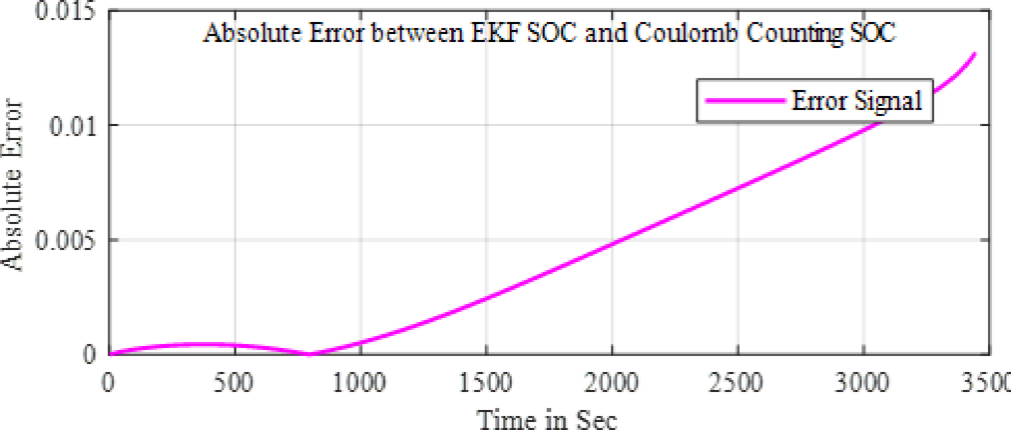
Error between the two methods.

**Fig. 8:**
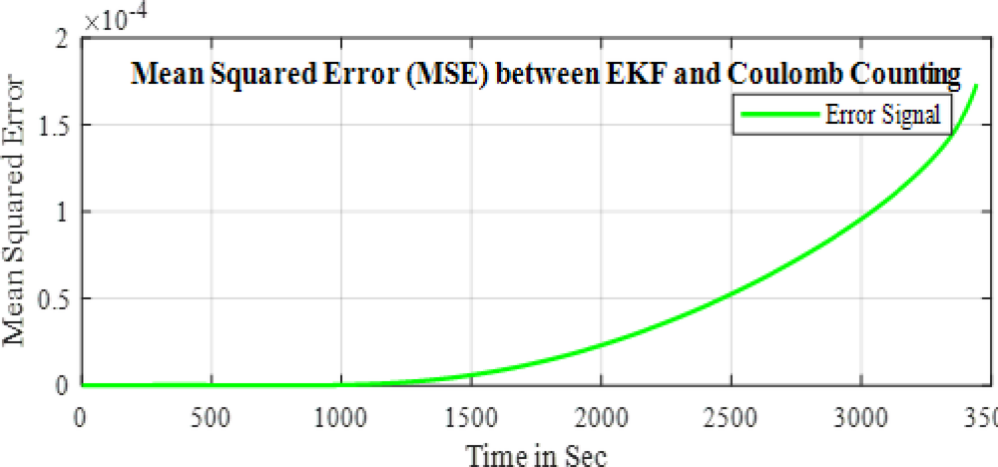
MSE between the two methods.

In the configuration window, set the test parameters, defining high-current pulses lasting a few seconds, followed by rest phases to allow the battery to stabilize before the next pulse. You can adjust the number of cycles, pulse width, and rest durations to simulate real-world conditions effectively. HPPC tests are crucial for assessing a ’battery’s power and energy capabilities, as well as its internal resistance, derived from the voltage response to current pulses. After configuration, initiate the test by pressing ‘Start’ in PSTrace. The software manages the potentiostat to apply the programmed pulses while recording real-time voltage and current data. Once the test is complete, export the results to a CSV file for further analysis in Excels. This analysis provides a comprehensive understanding of the battery’s dynamic power performance and overall health, essential for optimizing battery applications in various fields. The Fig. 5 shows the static capacity test discharge curve at 1 C rate and the Fig. 6 shows the SOC estimation comparison of the EKF and Coulomb Counting method. The Figs. 7 and 8 shows the error and the Mean Square Error (MSE) between the two estimation methods.

**Fig. 9:**
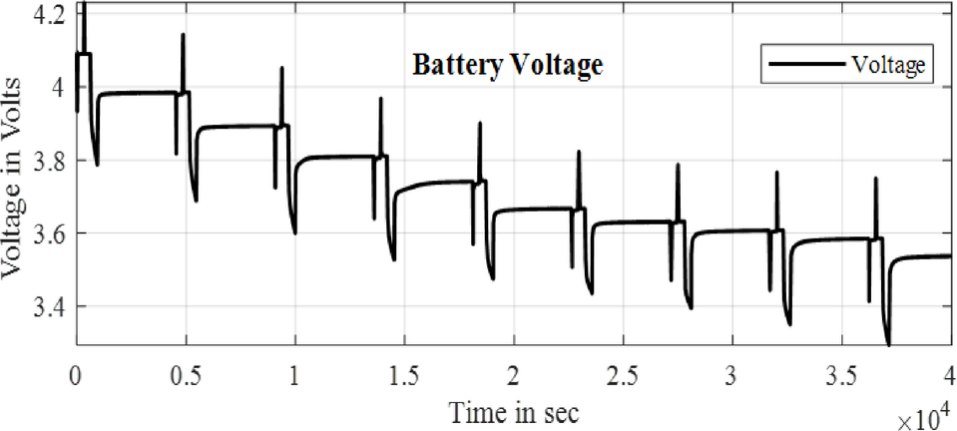
HPPC voltage response

**Fig. 10:**
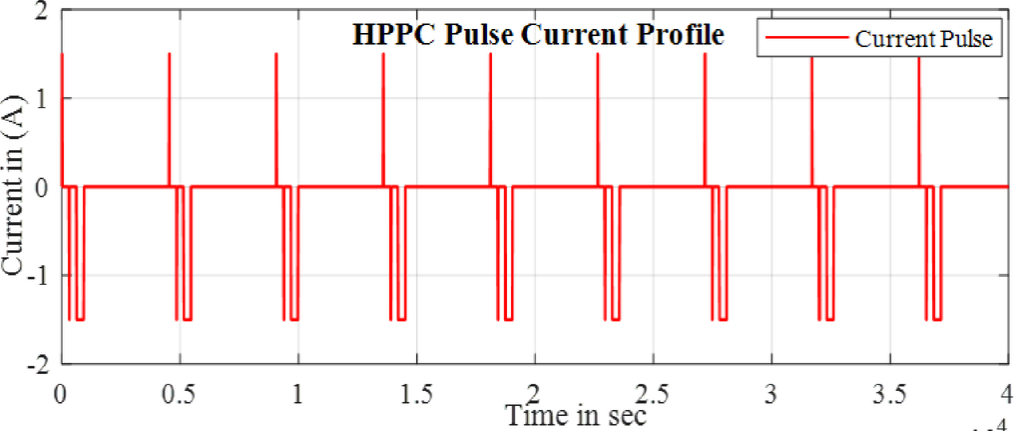
HPPC current profile.

**Fig. 11:**
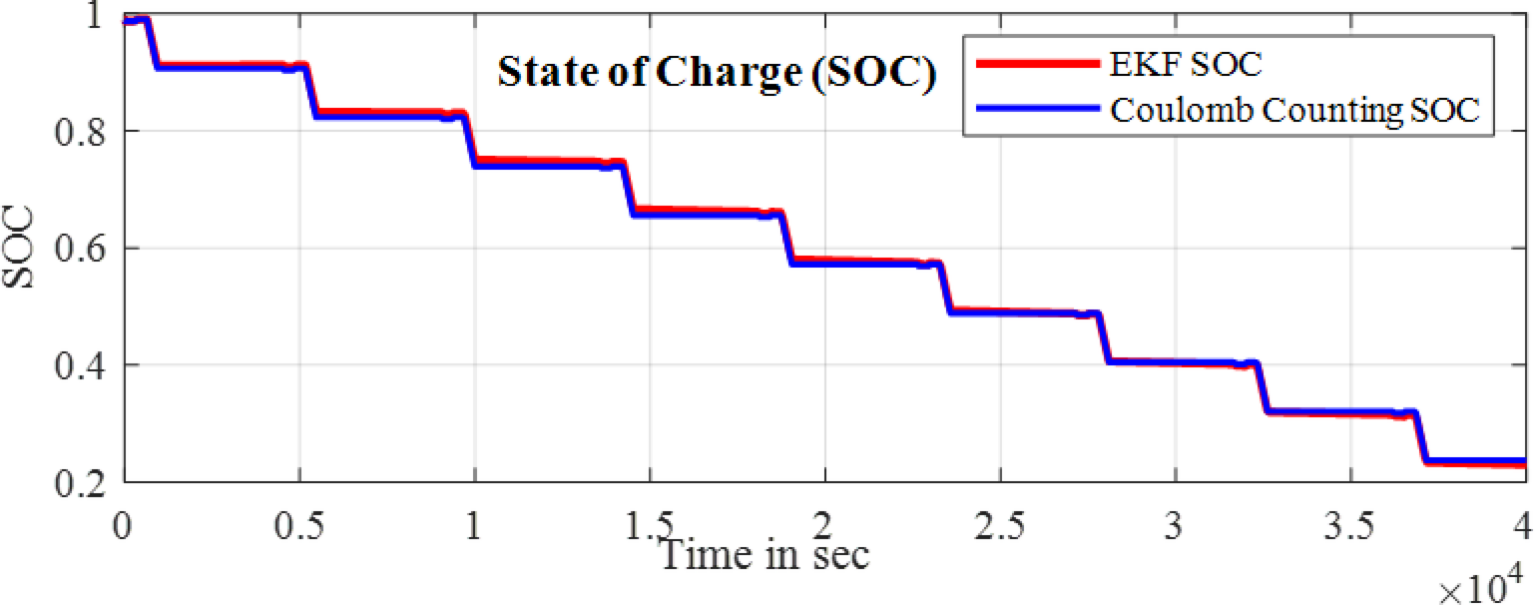
SOC estimated by EKF and CC methods.

Here the cell was tested with two different test one is static capacity test and second is using the HPPC test to validate the SOC estimation of the model. The Fig. 9 and 10 shows the HPPC test voltage response of the cell and the Pulse current Profile given to the model of the cell. Figure 11 Shows the SOC estimated by the EKF and CC methods for the HPPC pulse input with reduction of 10% SOC from 100 to 10%.

**Fig. 12:**
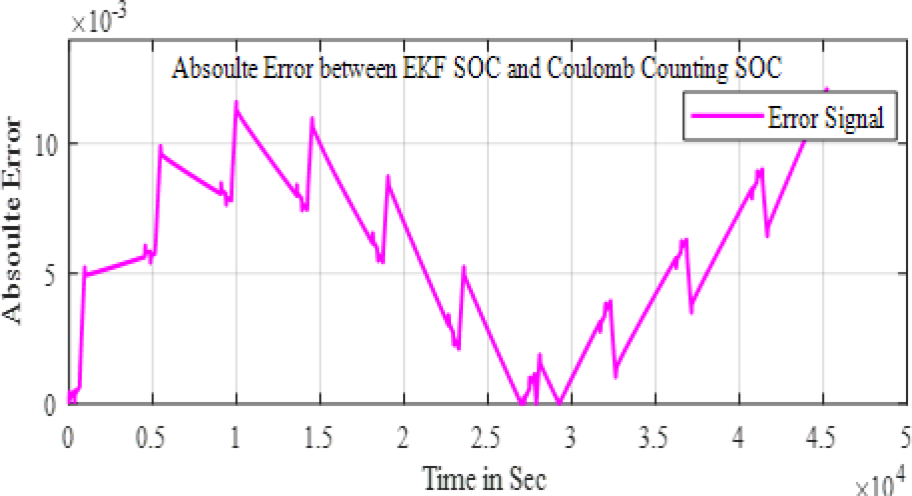
Error between the two methods.

**Fig. 13:**
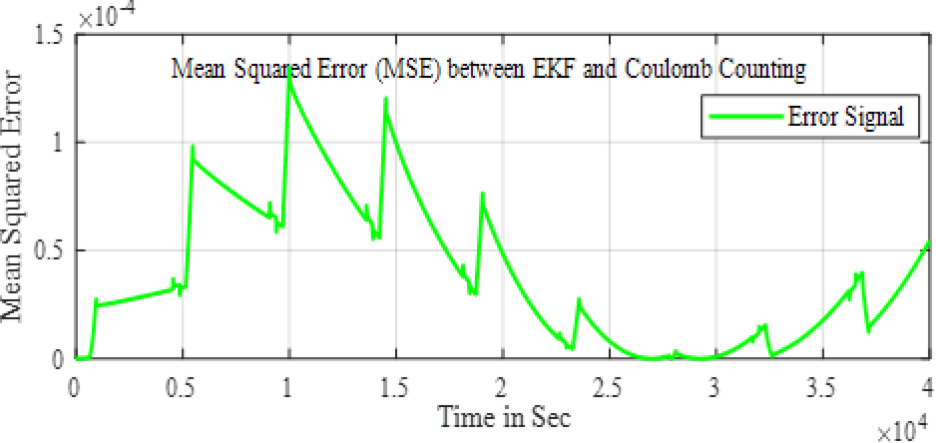
MSE between the two methods.

The Fig. 11 shows the comparison of the SOC estimation of the two different methods and the Figs. 12 and 13 show the error and MSE error between the two estimation methods.

**Fig. 14:**
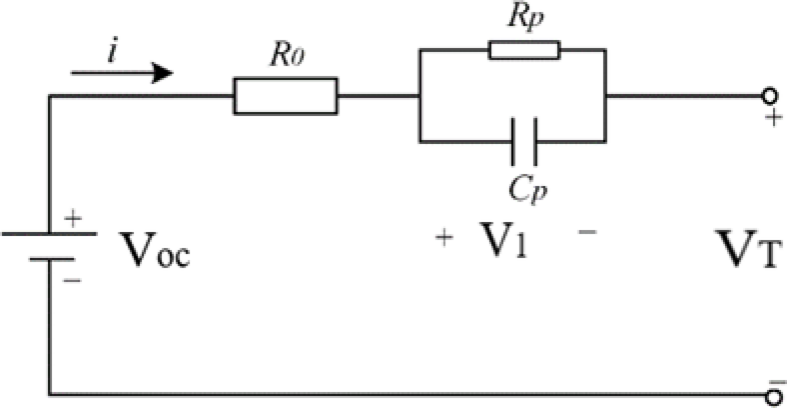
Thevenin circuit or equivalent circuit for our battery.

Then for finding SOC using Extended Kalman Filter first a block called parameter update has been made which has all the parameter like internal resistance (R0), polarization resistance (Rp), polarized capacitance (Cp) and open circuit voltage (Voc)^12^. Basically, this block helps to updated all the parameter values with respect to SOC with the help of the polynomial equation. Then those values are sent to the Thevenin Circuit block which has the equivalent circuit of our battery as shown in Fig. 14.

The single RC battery model simplifies the representation of battery behaviour by employing a resistor-capacitor (RC) circuit^13^. In this model, the battery is envisioned as an ideal voltage source connected in series with an internal resistance and a capacitor. The internal resistance, symbolized by the resistor, accounts for factors like material resistance within the battery and electrolyte resistance. Meanwhile, the capacitor embodies the ’battery’s charge storage capability, directly tied to its capacity. Estimating SOC becomes feasible through this model by measuring battery voltage and current, followed by employing estimation algorithms like the extended Kalman filter. This algorithm aids in estimating Q(t), facilitating the determination of the battery’s SOC^14^.

According to the results shown in Fig. 10, the difference between the SOC estimated by the Extended Kalman Filter (EKF) and the reference SOC calculated by coulomb counting would be nearly 3.8% without utilizing the updated electrical model for aged cells. This indicates that EKF’s effectiveness in SOC estimation significantly depends on the availability of an updated electrical model for batteries.

## Hardware implementation

### Circuit diagram

**Fig. 15:**
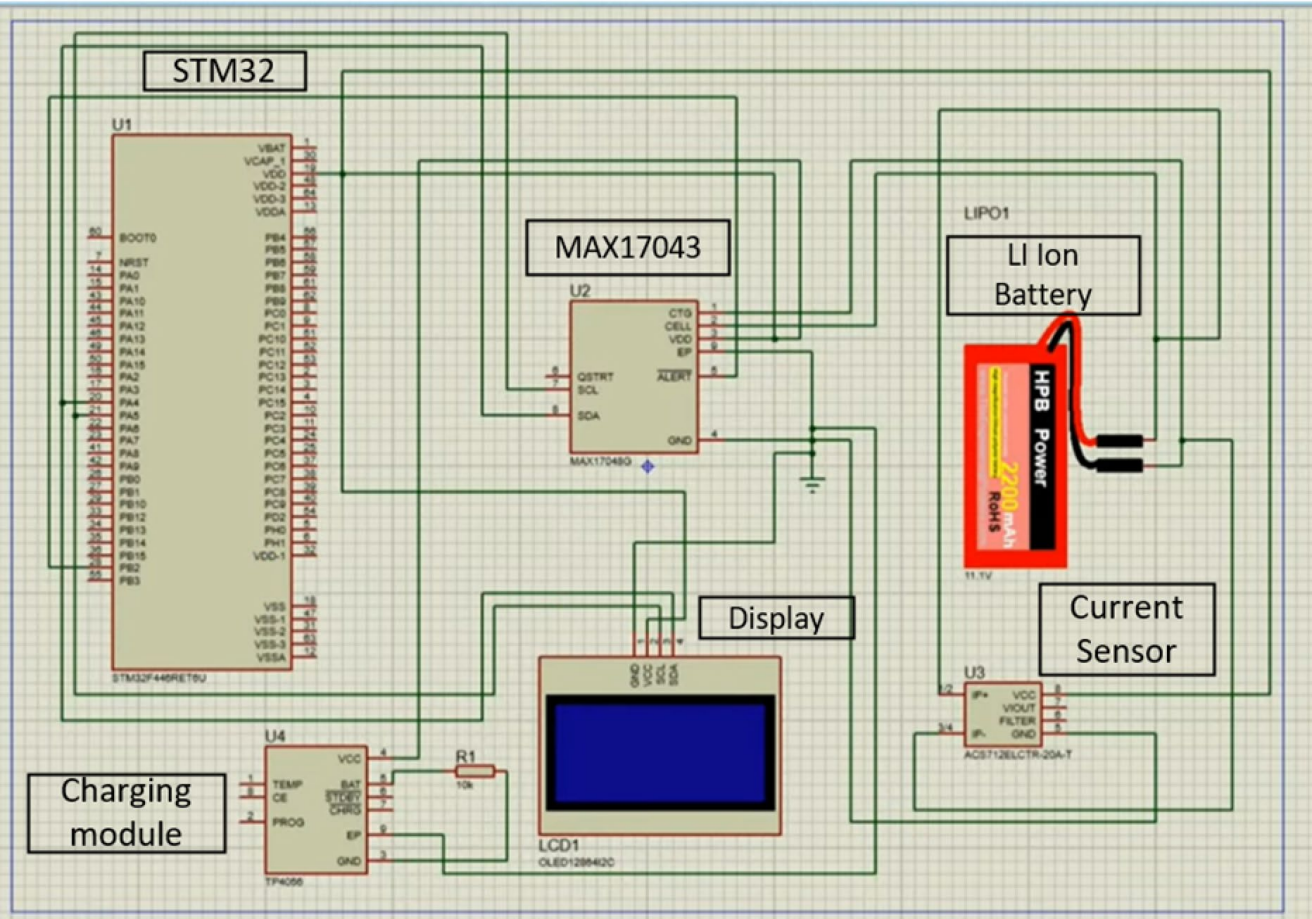
Proteus circuit diagram.

The circuit is drawn in the Proteus software shown in Fig. 15 by connecting logically using the bill of materials provided below in consideration to serve their own purposes individually.

### Hardware implementation

The optimal configuration is determined by identifying the set that minimizes the mean error value, which is then integrated into the new model. The process begins by discharging the cell at a constant current while measuring its terminal voltage, followed by calculating the voltage derivative to understand its behaviour over time. Reference points for SOC are established at 92% and 15%, serving as benchmarks for the voltage derivative. The optimization algorithm is then executed to update the model parameters based on these reference points. Finally, the refined model is incorporated into the Extended Kalman Filter (EKF), facilitating enhanced state estimation and improved performance monitoring of the battery cell. This comprehensive methodology ensures that the model accurately reflects the ’battery’s dynamic behaviour, optimizing its management and efficiency.

**Fig. 16:**
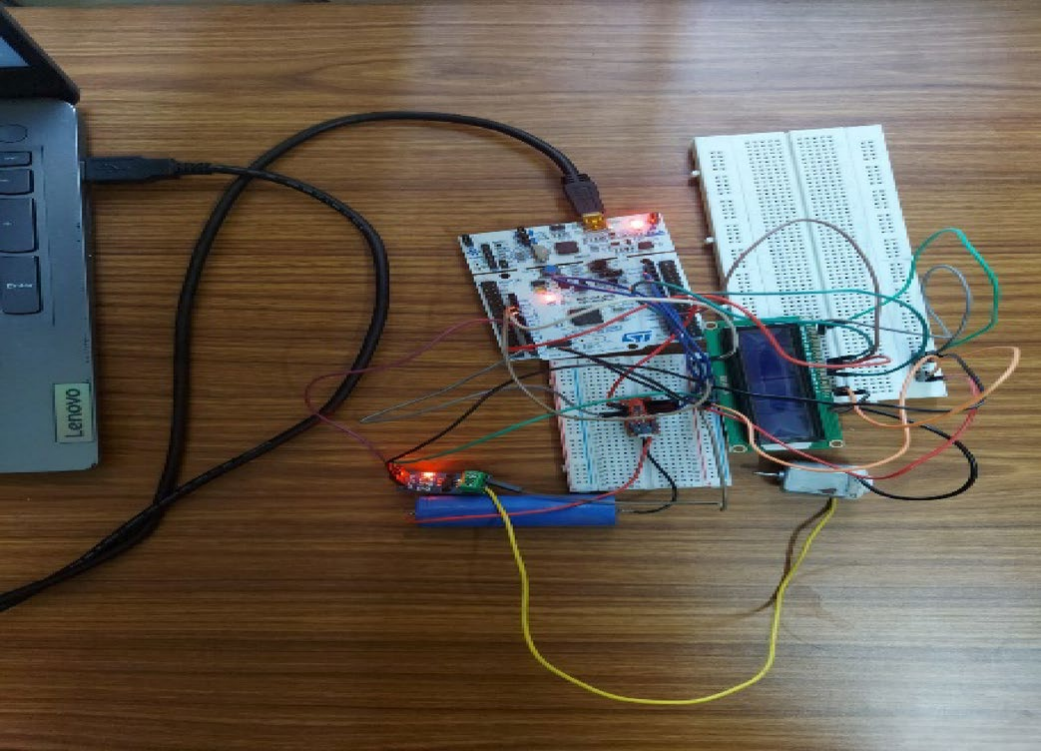
Hardware circuit.

**Fig. 17:**
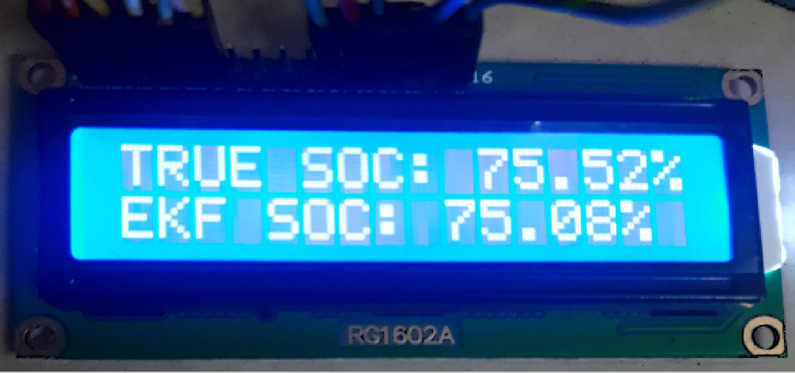
Hardware circuit.

A rechargeable Lithium-ion battery of form factor 18,650 has been chosen with ratings of 4.2 V (max) with 3.9 V (min) 1200mAh. The battery is put under the HPPC test to look into its discharge characteristics. A controller for processing the subject battery’s state and predicting the output needs to be chosen. The microcontroller Arduino Uno is selected initially to prototype the system and later tried with STM32 F446RE microcontroller for better performance^15^. Figures 16 and 17 shows the hardware setup consists of the STM board has similar form factors to Arduino Uno and can accommodate its expansion connectors, with multiple options for IDE’s and on-board debugger/programmer the board stands out in its processing capabilities. The STM32F446RE provides from 256-Kbyte to 512-Kbyte Flash memory, 128-Kbyte SRAM and from 64 to 144 inbuilt pins in packages as small as 3.85 × 3.728 mm.

Different modules have been imported to the software to provide for the Microcontroller and battery models to be simulated. This circuit design helps in visualizing and implementing the physical circuit later without damaging the components as the battery is prone to short circuit and the low current carrying capacity modules like the MAX17043 sensor and even the microcontroller if current leaks from a larger battery through the sensor are susceptible to damage or melting^16^. Here the Table 1 shows the specifications of the cell tested for the research work.

**Table 1:** Cell specifications.

S. no	Parameters	Specification
1	Cell type	Samsung ICR18650-26 F
2	Capacity	2.6Ah
3	Discharge voltage	2.75 V
4	Charge voltage	4.2 V
5	Nominal voltage	3.7 V
6	Cell dimension	Height : 65.00 mm max Diameter : 18.40 mm max
7	Operating temperature	Charge : 0 to 45 ℃ Discharge: -20 to 60 ℃

**Fig. 18:**
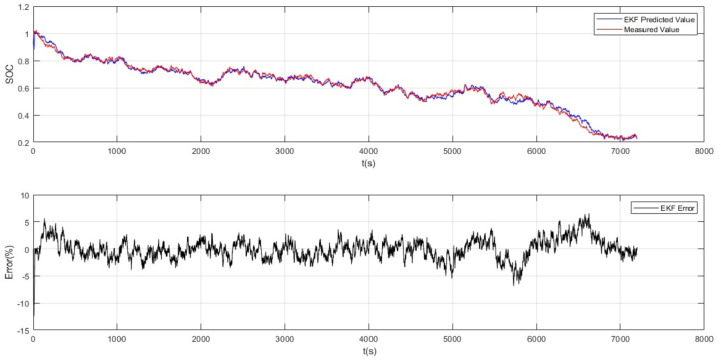
Hardware result – SOC estimation and error between the two methods.

**Fig. 19:**
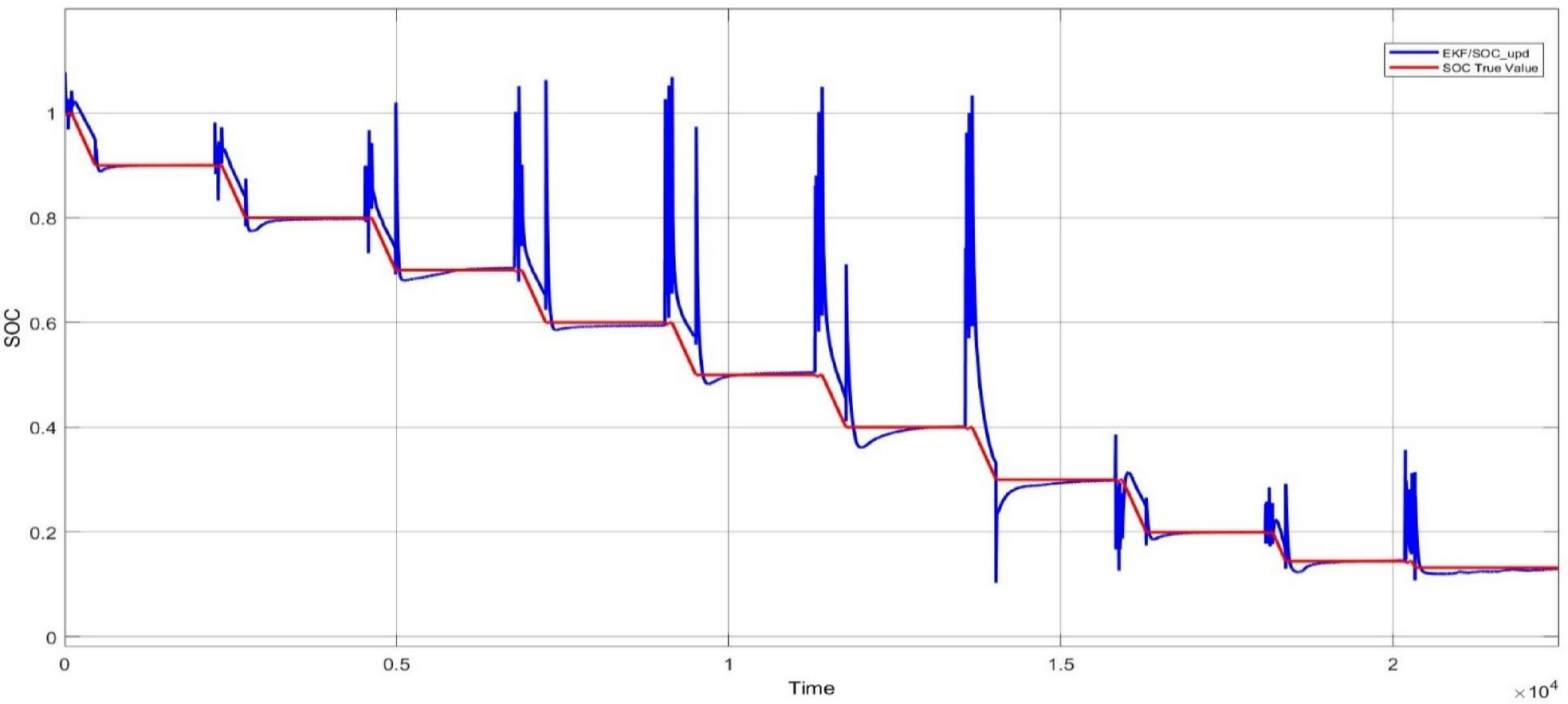
Hardware –result - EKF based SOC estimation with coulomb counting method.

**Fig. 20:**
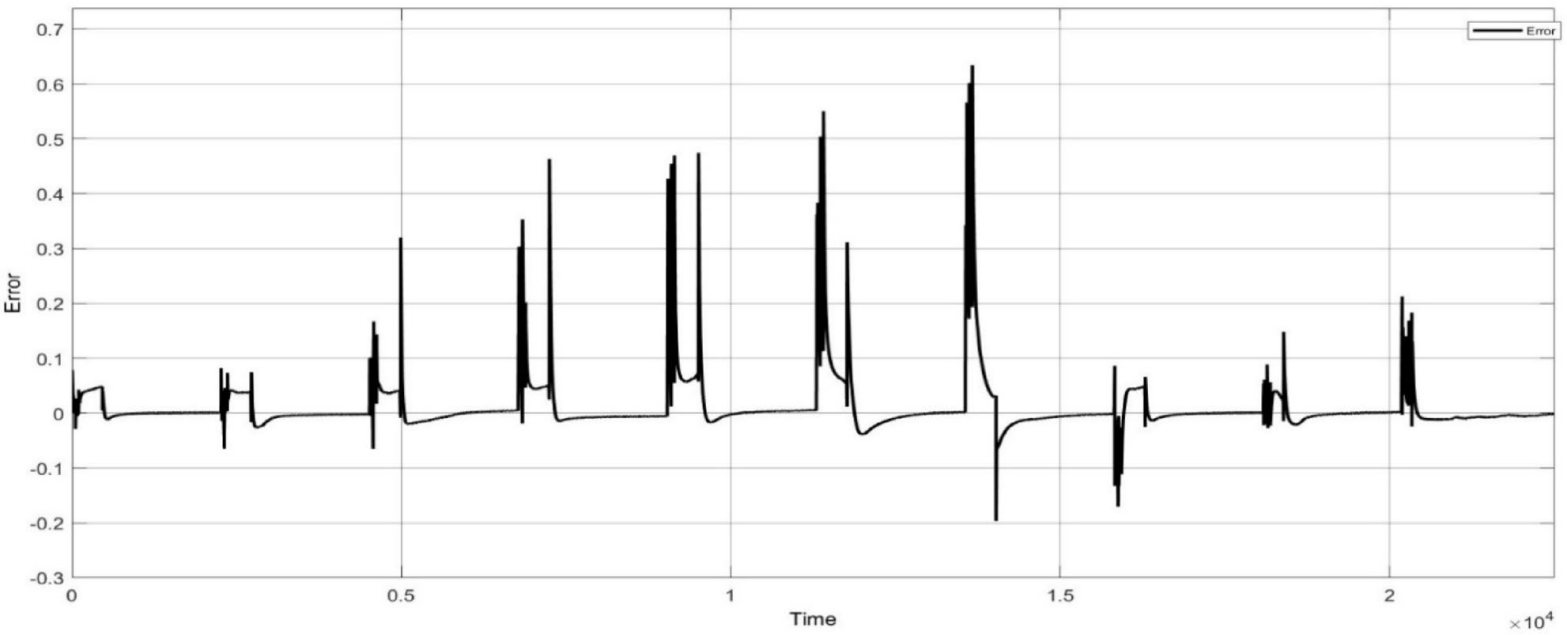
Error between the EKF based SOC estimation and coulomb counting method.

With the hardware model the cell is connected to the 5 V DC motor and tested to estimate the SOC from 100 to 10% and the same was compared with the measured value and show in Fig. 18. To validated the Hardware reliability the model was tested with the HPPC test and the Figs. 19 and 20 shows the SOC estimation result and the error between the two methods.

The sensor MAX17043 provides current, voltage and SOC readings of the battery as inputs to be fed into the microcontroller to process the data and provide predicted SOC as the output to be displayed in the 2.4” TFT display module. The MAX17043/44 packages are low cost, compact fuel-gauge systems for host side systems using lithium-ion batteries. It uses a battery modelling scheme called as Model Gauge to calibrate and track battery’s SOC continuously over a wide varying charge/discharge profile.

The display module can be customised to provide the readings in an attractive and intuitive method. The SOC can be represented using a bar or curve with percentage indication in numbers and colour coding in graph to immediately capture the user’s attention on the depletion level. The levels from 60 to 100% in green, 30–60% in yellow, 15–30% in orange and below 15% in red with a blinker to indicate the driver. A 5 V DC motor is used as a load simulator to consume battery capacity manually and test different levels of SOC. Charging module can remove the stored capacity apart from charging the battery back. Battery holder, SJT connectors and jumper cables with breadboard are used to connect and keep the components tightly packed to prevent errors in measurement due to vibration of the components.

A 16/2 LCD display can be also used to display the SOC output for end user. The above-mentioned materials are to be scaled as per the size of the battery being considered and the use case its serving, although the microcontroller is sufficient for the SOC algorithm implementation regardless of the battery size. The setup programmed uses the same logic implemented in MATLAB code with vectors and time functions in addition to account for the real-life model. The integration of the chosen components – the Lithium-ion battery, the MAX17043 sensor, microcontrollers STM32F446RE, TFT display module, and additional load simulator – for monitoring and predicting SOC of the battery has provided a robust platform for battery management and testing. The selection of components was based on their compatibility, performance, and cost-effectiveness.

Throughout the testing process, the system exhibited promising results in accurately tracking the ’battery’s SOC using the Model Gauge battery modelling scheme implemented by the MAX17043 sensor. The microcontroller, especially the STM32F446RE, offered ample processing capabilities to handle the data from the sensor and execute the SOC prediction algorithm efficiently. The customized display module, featuring intuitive representations of SOC levels through graphical indicators and colour coding, proved effective in providing users with clear insights into the ’battery’s depletion status. Additionally, the inclusion of a load simulator facilitated manual testing of various SOC levels, enhancing the system’s versatility in real-world scenarios.

Despite the overall success of the system, a notable observation was an error margin of approximately 4.2% in SOC prediction. This discrepancy, while relatively small, could potentially impact the accuracy of battery management decisions, especially in critical applications. Further analysis and optimization of the SOC prediction algorithm may be necessary to minimize such errors and enhance the system’s reliability. Overall, the developed battery management system demonstrates significant potential for various applications requiring precise monitoring and management of Lithium-ion batteries. Further refinements and optimizations could elevate its performance and ensure more accurate SOC predictions, ultimately enhancing the system’s effectiveness and reliability in practical use cases.

#### Comparison of computational complexity

The Table 2 presents a comparison of different SOC estimation methods based on their accuracy, speed, complexity, and real-time applicability. Coulomb Counting (CC) is the fastest but suffers from accuracy issues due to drift. Open-Circuit Voltage (OCV) provides high accuracy but is slow and impractical for real-time applications since the battery must be at rest. Extended Kalman Filter (EKF) offers high accuracy and is suitable for real-time applications, but it is more computationally intensive than CC or OCV. Unscented Kalman Filter (UKF) provides the highest accuracy for non-linear systems but is the slowest and most complex, making it less ideal for systems with limited processing power.

**Table 2:** Comparison of computational complexity.

Method	Accuracy	Speed	Complexity	Real-time applicability
Coulomb counting	Low (drift over time)	Fast (simple)	Low	Suitable for basic systems
Open-circuit voltage	High (at rest)	Slow (requires rest)	Low	Not suitable for real-time
EKF	High (dynamic)	Moderate (prediction-correction cycle)	High (modelling & filtering)	Suitable for real-time
UKF	Very high (non-linear systems)	Slow (non-linear handling)	High (complex filtering)	Suitable for real-time (but slower)

**Table 3:** Comparison of computational efficiency.

Performance measurement	EKF (extended Kalman filter)	UKF (unscented Kalman filter)
Time complexity	O(n^3^) cubic time complexity	O(n^3^) with higher constant factors for sigma point propagation
Model	Locally linear	Non-linear
Computational complexity	Slight nonlinear real time systems	Medium nonlinear systems
Operations	• Linearization of non-linear models using Jacobian Matrix computation. • Matrix multiplications for stepwise prediction and correction.	• Selection and propagation of sigma point. • Computing weighted mean and covariance
Key factor	Computationally efficient	Accuracy
Limitations	Linearization leads to inaccuracy with highly non-linear system causing divergence.	Computational burden over very high dimensional systems.
RMSE (root mean square error)^16^	0.1532	0.1531
Mean absolute error^16^	0.0856	0.0859
Max error^16^	0.8026	0.8027
Computational time^17^	266.13 microseconds	3294.2 microseconds

Overall UKF provides a system with better accuracy, more stability and computationally expensive system compared with EKF but citing the cost and complexity of UKF over EKF it is less preferrable in real time application of BMS. The quantitative comparison of computational efficiency for EKF & UKF are presented in Table 3. UKF shall be put into use in much complicated systems like that of satellite tracking where accuracy is of utmost importance over computation cost.

## Conclusions

In contrast to the conventional Coulomb counting approach, the implemented work effectively illustrated how the Extended Kalman Filter (EKF) algorithm addresses non-linearity difficulties and achieves quicker, more accurate SOC estimates. Both SCT and HPPC tests were performed using the Samsung ICR18650-26 F 2.6Ah, 3.7 V lithium-ion battery. With an error difference of less than 2% between the SOC estimate findings from EKF and Coulomb counting, the HPPC test made it possible to construct and simulate a 1RC battery model. The MAX17043 sensor and STM32F446RE microcontroller were used in a hardware configuration that successfully evaluated SOC of the battery while feeding a DC motor load using an EKF-based technique, with an inaccuracy of less than 4% when compared to the real SOC. For long-term data processing on a server and real-time SOC monitoring, the created hardware design may be combined onto a single PCB and improved with Wi-Fi connection. Integration of the suggested method with battery management systems (BMS) for electric vehicles (EVs) and other applications that use rechargeable lithium-ion batteries has a lot of promise. The solution may improve battery longevity and efficiency, decrease e-waste from premature disposal, and meet the increasing demand in the EV dashboard and rechargeable battery industries by offering precise SOC predictions and allowing real-time updates.

However, in the proposed work it is not considered the impact of different C-rate in this model. The model is derived from experiments conducted with small Li-ion cells, but its applicability to larger cells remains to be verified. Additionally, all experiments were conducted at room temperature. The drawbacks present in this method is that EKF has approximation errors and underestimates state uncertainties, also is computationally complex for the developers. To enhance the model’s universality, investigating the effects of different C-rate and temperature on the hybrid electrical model would be valuable. Furthermore, exploring how these factors influence the model’s adaptive algorithm could be a promising avenue for further research. Large battery packs pose additional challenges for state-of-health (SOH) estimation due to cell imbalances. Thermal management of the entire pack also plays a role in this issue. Addressing thermal management concerns could involve developing a sophisticated 3D thermal model at the pack level, considering uniform cell temperature distribution and the influence of neighbouring cells.

## Data Availability

The datasets used and/or analysed during the current study available from the corresponding author on reasonable request.
